# Indirect effects of social support and hope on quality of life *via* emotional distress among stroke survivors: A three-wave structural equation model

**DOI:** 10.3389/fpsyt.2022.919078

**Published:** 2022-07-28

**Authors:** Ted C. T. Fong, Temmy L. T. Lo, Rainbow T. H. Ho

**Affiliations:** ^1^Centre on Behavioral Health, Faculty of Social Sciences, The University of Hong Kong, Hong Kong, Hong Kong SAR, China; ^2^Department of Social Work and Social Administration, Faculty of Social Sciences, The University of Hong Kong, Hong Kong, Hong Kong SAR, China

**Keywords:** depression, functional impairment, longitudinal, moderated mediation, perceived stress, stroke-specific quality of life

## Abstract

**Objectives:**

Stroke survivors are prone to functional impairments and reduced quality of life (QoL). This study examined the mediating role of social support and hope in the relationships between functional impairment, emotional distress, and QoL.

**Methods:**

A total of 188 stroke survivors in Hong Kong completed assessments on functional impairment, social support, and hope at baseline, with follow-up measurements of emotional distress at 2 months and QoL at 8 months. Structural equation modeling with bootstrapping was used to analyze the direct and indirect effects of functional impairment on emotional distress and QoL *via* the mediating factors of social support and hope.

**Results:**

The partial cascading model provided an adequate fit to the data. Functional impairment had significant negative direct effects on hope and physical QoL and social support had significant positive direct effects on hope and physical QoL. Social support had a significant positive indirect effect on physical QoL *via* hope and perceived stress and on psychosocial QoL *via* hope and depression. Functional impairment and hope had a significant negative interaction effect on perceived stress.

**Conclusion:**

The findings support a mediating role for hope in the relationship between social support and QoL in stroke survivors. The protective effect of hope on perceived stress was stronger among patients with greater functional impairment.

## Introduction

Stroke is a neurovascular disease that places a considerable health burden on the patient and has a substantial economic cost to society (1). Stroke survivors often suffer from functional impairment in multiple domains, emotional distress in terms of perceived stress and depressive symptoms, and poor quality of life (QoL) (2). Given the increasing prevalence of stroke and its associated disability burden (3), it is important to identify protective factors for the long-term recovery of stroke survivors. Social support is a potential protective factor that might facilitate psychosocial transition and contribute to a better prognosis for the rehabilitation of stroke survivors. The presence of physical disabilities such as impaired mobility, speech, cognition, and vision probably decreases survivors’ abilities to communicate and interact with other people. This could, in turn, lead to reduced social connection with family members and friends, resulting in decreased social support and increased social isolation for stroke survivors.

Systematic reviews (4, 5) have found associations of poor social support with depression, reduced QoL, and worse physical recovery among stroke survivors. Perceived social support has shown significant temporal associations with greater community participation (6), lower depression levels (7), and better functional status following stroke onset (8, 9). Among stroke patients, social support has been found to mediate the effect of functional ability on depression and QoL (10), and anxiety and depressive symptoms have been found to mediate the relationship between social support and sleep quality (11). However, most of the studies of stroke survivors were limited by a cross-sectional design (10, 12–14) or a small sample size (*N* < 50) (8, 9, 15, 16). Longitudinal studies with larger sample sizes are needed to examine the mediating role of social support in stroke recovery.

Hope could be an essential coping strategy and a psychological resource for patients with chronic illness, including stroke survivors. Most relevant studies (17–24) have adopted a qualitative approach to investigate the relevance of hope in the perspectives and recovery experiences of stroke survivors. A narrative review (22) found that social support from friends and family members could be a potential factor in generating hope in stroke patients. In particular, social support can be a source of motivation and encouragement during the rehabilitation process, helping to foster or maintain a sense of hope among stroke survivors.

Regarding quantitative studies, previous psychometric studies (25, 26) have found satisfactory factorial validity and reliability for the Dispositional Hope Scale in measuring hope in stroke survivors. A cross-sectional study (27) found a moderate positive correlation between hope and QoL among 40 stroke survivors with moderate to severe functional impairment. A recent randomized controlled trial (28) showed that a hope intervention improved the level of hope and enhanced the functional ability of 94 stroke survivors in China. However, few longitudinal studies have investigated the protective role of hope in the recovery process of stroke survivors.

To our knowledge, no empirical studies have examined the relationship between social support and hope or the potential mediating role of hope in improving the functional outcomes of stroke survivors. In view of the research gaps, the first objective of the present study was to evaluate the temporal effects of social support and hope on emotional distress (perceived stress and depressive symptoms) and functional outcomes (physical and psychosocial QoL) of stroke survivors. The second objective was to investigate the potential mediating roles of hope in the relationship between social support and the emotional distress and QoL of stroke survivors. This examination aimed to elucidate the indirect effects of social support on emotional distress and functional outcomes *via* hope. Clarifying the potential interplay between social support and hope (as psychosocial coping strategies) and physical QoL *via* emotional distress could contribute to a better understanding of the mind–body interaction in the recovery process of stroke survivors.

## Materials and methods

### Procedures and participants

The present study adopted a longitudinal study design with three assessments across 8 months: baseline (Time 0), a 2-month follow-up (Time 1), and an 8-month follow-up (Time 2). The participants were recruited using convenience sampling through community rehabilitation centers and hospitals, patient self-help groups, and directly from the community in Hong Kong. Two trained research assistants screened 209 patients for eligibility based on the following inclusion criteria: (1) a diagnosis of ischemic or hemorrhagic stroke; (2) symptoms ranging from having no significant disability, despite experiencing residual symptoms, to those with moderately severe disability [modified Rankin Scale (mRS) scores of 1–4]; and (3) aged between 18 and 64 years. Ten patients did not fulfill the inclusion criteria or were excluded because they were mini-stroke cases or aged above 65 or had a MRS score of 5. Ten patients refused to participate in the study because of time constraints. One patient died before the baseline assessment. All participants provided written informed consent before completing the baseline assessment. The participants were given a financial incentive of HKD 150 (USD 20) for completing the three assessment waves. Ethics approval was obtained from the Human Research Ethics Committee of the authors’ university (EA1702058).

A total of 188 stroke survivors aged less than 65 years voluntarily took part in the study at Time 0 at which point they completed a self-report questionnaire on social support, hope, perceived stress, depression, and stroke-specific QoL. The average age of the participants was 55.2 years [standard deviation (SD) = 7.82]. Approximately 60% of the participants were male, married, had received at least 10 years of education, and reported a diagnosis of ischemic stroke. The mean duration since stroke onset was 3.76 years (SD = 4.22). At Time 1, 175 participants completed the follow-up assessment on perceived stress and depression. At Time 2, 123 participants remained in the study and completed the follow-up assessment on stroke-specific QoL measures as distal outcomes. The longer time lag (6 months) between Time 1 and Time 2 was designed to examine the longer-term functioning of the stroke survivors.

### Measures

The study adopted the simplified mRS to clinically diagnose the degree of functional impairment of the stroke survivors (29). The participants were classified into one of seven levels of disability, where 0 indicated no symptoms and 6 indicated that the patient had died. In the present study, only those with no significant disability but with symptoms (mRS score = 1), slight disability (mRS score = 2), moderate disability (mRS score = 3), or moderately severe disability (mRS score = 4) were included.

The 12-item Multidimensional Scale of Perceived Social Support (30) and the 6-item Adult State Hope Scale (31) were, respectively used to assess the level of social support and hope perceived by the participants. The items on perceived social support and hope were, respectively rated on a 7-point Likert scale (from 1 = “very much disagree” to 7 = “very much agree”) and an 8-point Likert scale (from 1 = “definitely false” to 8 = “definitely true”). There were three subscales on perceived social support from family, friends, and a significant other. The total scores for social support and hope had theoretical ranges from 1 to 7 and from 8 to 48, respectively. Both scales showed good levels of internal consistency (α = 0.91–0.93) at Time 0.

The 10-item Perceived Stress Scale (32) and the 7-item depression subscale of the Hospital Anxiety and Depression Scale (33) were used to measure the level of subjective stress and depressive symptoms, respectively, in the past 2 weeks as appraised by the participants. The items of the Perceived Stress Scale and the Hospital Anxiety and Depression Scale were answered using 5-point and 4-point Likert scales, respectively. The perceived stress score ranged from 0 to 40, and the depression score ranged from 0 to 21. Acceptable levels of internal consistency (α = 0.73–0.78) were found for these two measures across Time 0 and Time 1.

The 12-item short form of the Stroke-specific Quality of Life Scale was used to assess the participants’ QoL (34). The instrument inquired about the extent of interference in stroke-specific domains such as mobility, self-care, upper extremities, and language. The items were rated on a 5-point Likert scale from 1 = “completely agree” to 5 = “completely disagree,” where a higher score indicated better functioning. The 7-item physical QoL subscale score ranged from 7 to 35 and the 5-item psychosocial QoL subscale score ranged from 5 to 25. Both subscales displayed acceptable levels of internal consistency (α = 0.72–0.81) across Time 0 and Time 2.

### Data analysis

Attrition analysis was conducted to investigate the presence of attrition bias owing to those who dropped out of the study over the 8-month study period. Missing data were handled through the missing-at-random assumption (35). Structural equation modeling using the robust maximum likelihood estimator in Mplus 8.6 (36) was conducted to estimate the direct and indirect effects of social support and hope on stroke-specific QoL *via* emotional distress. None of the main study variables deviated from the normal distribution; the range of skewness was −0.87–0.53, and the range of kurtosis was −1.08–1.30. Perceived social support was posited as a latent factor measured by the three subscales of social support from family, friends, and a significant other. Hope was modeled as a latent factor measured by six items. The structural equation model (SEM) was used to estimate the effect of perceived social support on hope in stroke survivors at Time 0.

The latent factors of social support and hope at Time 0 were hypothesized to be predictors of stroke-specific QoL (both physical and psychosocial QoL) at Time 2. The observed variables of perceived stress and depression at Time 1 were hypothesized to be mediator variables. The baseline assessments of emotional distress and QoL were included in the SEM to account for autoregressive effects as a measure of the stability of individual differences over time. The SEM control variables were sex, age, number of cohabitants, type of stroke, and onset time of stroke event. The model fit was evaluated *via* the approximate fit indices based on the following cutoff criteria (37): comparative fit index and Tucker–Lewis index ≥ 0.95; root mean square error of approximation and standardized root mean square residual ≤ 0.06. Three nested SEM models were applied—a combined model, a partial cascading model, and a cascading model—which estimated full, partial, and no direct effects, respectively, from Time 0 predictors on Time 2 outcomes. The fits of the nested models were compared using the scaled chi-square difference test. Model parsimony was evaluated using Bayesian information criterion (BIC), with a difference of at least 10 denoting a substantially better fit for the model with a smaller BIC.

To account for the probable skewed distribution, the indirect effects of social support and hope at Time 0 on QoL at Time 2 *via* perceived stress and depressive symptoms at Time 1 were estimated with 10,000 bootstrap draws. The indirect effects were regarded as statistically significant if the 95% bootstrapped confidence interval (CI) excluded zero. The potential moderating role of functional impairment was examined by modeling the latent interaction terms between functional impairment and latent factors (social support and hope). Latent interaction analysis was used to evaluate the effects of mRS score × social support on hope and of mRS score × hope on perceived stress and depression at Time 1. The interaction effects were probed by plotting graphs of simple effects across the degree of functional impairment. The R-square value indicated the proportion of explained variance of the outcome variables.

## Results

### Sample profile and attrition analysis

Table 1 displays the descriptive statistics, bivariate correlations, and reliability of the study variables. Half (51%) of the sample reported slight disability, followed by moderate disability in 25%. Functional impairment was negatively associated with hope and both physical and psychosocial QoL (*r* = −0.21 to −0.46, *p* < 0.01) and positively associated with perceived stress and depression (*r* = 0.19 to 0.31, *p* < 0.01). Social support was moderately positively correlated with hope (*r* = 0.53, *p* < 0.01). Both social support and hope were negatively associated with perceived stress and depression (*r* = −0.22 to −0.59, *p* < 0.01) and positively associated with physical and psychosocial QoL (*r* = 0.25 to 0.47, *p* < 0.01). Perceived stress and depression were negatively associated with physical and psychosocial QoL (*r* = −0.35 to −0.61, *p* < 0.01).

**TABLE 1:** Descriptive statistics, bivariate correlations, and reliability of the study variables across Time 0 and Time 2.

	M (*SD*)	Range	1	2	3	4	5	6	7	8	9	10	11	12	13	14	15	16
1. Male	0.62 (0.48)	/	/															
2. Age	55.2 (7.82)	23 – 64	0.05	/														
3. No. of co-residents	2.09 (1.20)	0 – 5	–0.05	–0.10	/													
4. Onset time (years)	3.76 (4.22)	0 – 25	0.01	0.14	–0.17	/												
5. Ischemic stroke	0.61 (0.54)	/	0.02	0.14	–0.09	–0.24	/											
6. T0 mRS	2.10 (0.77)	1 – 4	−0.31*	–0.04	0.20*	–0.02	–0.18	/										
7. T0 Social support	4.58 (1.45)	1 – 7	–0.12	–0.01	0.01	–0.02	–0.03	–0.09	*0.93*									
8. T0 Hope	33.6 (9.00)	6 – 48	–0.04	0.02	–0.01	–0.07	0.09	−0.21*	0.53*	*0.91*								
9. T0 Perceived stress	19.4 (5.38)	0 – 40	–0.07	–0.08	0.05	–0.02	–0.13	0.19*	−0.32*	−0.49*	*0.76*							
10. T1 Perceived stress	19.4 (5.51)	0 – 40	–0.21	–0.18	0.09	–0.11	–0.05	0.13	−0.22*	−0.41*	**0.63** *	*0.78*						
11. T0 Depression	7.25 (3.87)	0 – 21	–0.09	–0.12	0.12	0.00	–0.10	0.31*	−0.41*	−0.59*	0.63*	0.53*	*0.73*					
12. T1 Depression	6.59 (3.85)	0 – 21	0.03	−0.18*	0.05	–0.08	–0.13	0.25*	−0.35*	−0.50*	0.55*	0.58*	**0.68** *	*0.77*				
13. T0 Physical QoL	23.1 (6.12)	7 – 35	0.16	0.11	−0.26*	–0.03	0.24*	−0.46*	0.25*	0.34*	−0.39*	−0.36*	−0.54*	−0.35*	*0.80*			
14. T2 Physical QoL	23.8 (5.97)	7 – 35	0.11	0.17	–0.24	–0.05	0.17	−0.40*	0.39*	0.43*	−0.58*	−0.45*	−0.57*	−0.53*	**0.69** *	*0.81*		
15. T0 Psycho QoL	16.4 (4.35)	5 – 25	0.26*	0.09	−0.21*	–0.07	0.24*	−0.35*	0.26*	0.44*	−0.48*	−0.42*	−0.61*	−0.35*	0.71*	0.64*	*0.72*	
16. T2 Psycho QoL	17.3 (4.49)	5 – 25	0.24	0.12	–0.16	–0.12	0.17	−0.32*	0.41*	0.47*	−0.58*	−0.48*	−0.56*	−0.55*	0.59*	0.78*	**0.73** *	*0.76*

Cronbach’s alpha are presented in italic at the diagonal; *p < 0.01; lagged correlations are bolded. mRS, modified Rankin Scale; Psycho QoL, Psychosocial quality of life.

There were no significant changes (Cohen *d* = 0.01–0.15, *p* = 0.10–0.97) in the levels of perceived stress and physical QoL of the participants in the study period. However, the participants reported significant decreases and increases (*d* = 0.23–0.29, *p* < 0.01) in depressive symptoms and psychosocial QoL, respectively. The attrition analysis did not reveal significant differences (*p* = 0.15–0.97) between the study completers (*N* = 123) and dropouts (*N* = 65) for any demographic characteristics, clinical characteristics, or study variables at baseline. The only exception was that the study dropouts displayed significantly lower levels of functional impairment (*d* = 0.16, *p* = 0.026) than the study completers at baseline.

### Structural equation model comparison and direct effects

Table 2 presents the fit indices of the specified SEMs. The cascading SEM showed a significantly worse fit (*p* < 0.05) than the other two models in theΔχ^2^ difference test. In the combined SEM, hope did not have a significant direct effect on the Time 2 QoL measures (*p* = 0.67–0.73), and the effects of the mRS score and social support on psychosocial QoL were not significant (*p* = 0.06–0.16). However, social support positively predicted Time 2 physical QoL [Unstandardized regression coefficient (*B*) = 0.70, standard error (SE) = 0.26, *p* < 0.01], and the mRS score negatively predicted Time 2 physical QoL (*B* = −1.00, SE = 0.48, *p* < 0.05). The Δχ^2^ difference test showed no significant difference (Δχ^2^(23) = 27.1, *p* = 0.25) between the partial cascading SEM and the combined SEM. Both models showed an acceptable fit to the data with similar model fit indices. However, the partial cascading model showed a substantially lower BIC and was more parsimonious than the combined SEM.

**TABLE 2:** Fit indices of combined and cascading structural equation models.

Model	Specification	χ^2^	df	RMSEA	CFI	TLI	SRMR	BIC	Δχ^2^ test (Δ df)	
1	Cascading	285.9*	184	0.054	0.944	0.928	0.063	17284	1 vs. 3	46.4* (28)
2	Partial cascading	267.1*	179	0.051	0.952	0.936	0.058	17291	1 vs. 2	20.4* (5)
3	Combined	239.6*	156	0.053	0.954	0.930	0.055	17383	2 vs. 3	27.1 (23)

*p < 0.05; χ^2^ = chi-square; df, degree of freedom; RMSEA, root mean square error of approximation; CFI, comparative fit index; TLI, Tucker–Lewis index; SRMR, standardized root mean square residuals; BIC, Bayesian information criterion; Δχ^2^ test = chi-square difference test.

### Results of the partial cascading structural equation model

**FIGURE 1:**
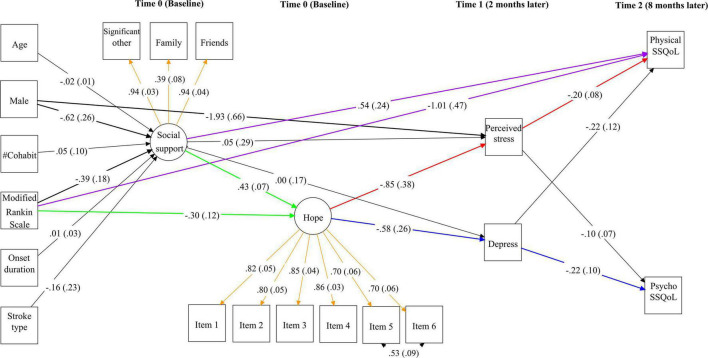
Unstandardized path coefficients from Time 0 social support and hope to Time 2 stroke-specific quality of life (SSQoL) *via* Time 1 perceived stress and depression in the partial cascading model. Significant paths among the study variables are highlighted in bold. Standard errors are shown in parentheses. The direct effects of functional impairment and social support on hope are highlighted in green and those on physical QoL are highlighted in purple. The indirect effects of hope on physical QoL *via* perceived stress and on psychosocial QoL *via* depression are marked in red and blue, respectively. Standardized factor loadings of social support and hope are shown in orange. For simplicity, the baseline measures of the QoL and psychological variables are not shown in this figure.

Figure 1 depicts the unstandardized path coefficients from Time 0 social support and hope to the Time 2 stroke-specific QoL measures *via* Time 1 perceived stress and depression in the partial cascading model. In the figure, the baseline measurements of physical and psychosocial QoL and psychological variables are omitted for ease of presentation. Significant factor loadings (orange in Figure 1) were found for the latent factors of social support (λ = 0.39–0.94, *p* < 0.01) and hope (λ = 0.70–0.86, *p* < 0.01). Age, number of cohabitants, type of stroke, and time since stroke onset did not show any significant effects (*p* = 0.13–0.78) on the main study variables. Men reported significantly lower levels of social support (*B* = −0.62, SE = 0.26, *p* = 0.017) and perceived stress (*B* = −1.93, SE = 0.66, *p* < 0.01) than women. Significant moderate autoregressive effects (*B* = 0.45–0.53, SE = 0.07–0.08, *p* < 0.01) were found for perceived stress, depression, and physical and psychosocial QoL across the repeated measurements.

The mRS score significantly negatively predicted social support (*B* = −0.39, SE = 0.18, *p* = 0.035) and hope (*B* = −0.30, SE = 0.12, *p* = 0.011). Social support had a significant positive effect on hope (*B* = 0.43, SE = 0.07, *p* < 0.01). After controlling for covariates and baseline measures, social support did not have a significant effect on perceived stress or depression (*B* = 0.00 to 0.05, SE = 0.17–0.29, *p* = 0.87–0.99). However, hope had a significant negative effect on perceived stress (*B* = −0.85, SE = 0.38, *p* = 0.025) and depression (*B* = −0.58, SE = 0.26, *p* = 0.026). Perceived stress significantly predicted lower physical QoL (*B* = −0.20, SE = 0.08, *p* = 0.012) but not psychosocial QoL (*B* = −0.10, SE = 0.07, *p* = 0.14). Conversely, depression significantly predicted lower psychosocial QoL (*B* = −0.22, SE = 0.10, *p* = 0.027) but not physical QoL (*B* = −0.22, SE = 0.12, *p* = 0.08). The model explained 5.7 and 32.7% of the variance in social support and hope at Time 0; 43.6 and 46.7% of the variance in perceived stress and depression at Time 1; and 57.7 and 59.3% of the variance in physical and psychosocial QoL at Time 2, respectively.

### Indirect effects of social support and hope on Time 2 quality of life measures

As shown in Table 3, hope had a significant positive indirect effect on physical QoL *via* Time 1 perceived stress (αβ = 0.169, 95% CI = 0.013–0.376) and on psychosocial QoL *via* Time 1 depression (αβ = 0.125, 95% CI = 0.003–0.345). These two indirect effects are marked in red and blue, respectively, in Figure 1. Similarly, social support had a significant positive indirect effect on physical QoL *via* hope and perceived stress (αβ = 0.074, 95% CI = 0.005–0.171) and on psychosocial QoL *via* hope and depression (αβ = 0.055, 95% CI = 0.001–0.148). The mRS score had a significant negative indirect effect on physical QoL *via* hope (αβ = −0.139, 95% CI = −0.286 to −0.029) and social support (αβ = −0.256, 95% CI = −0.621 to −0.002), and on psychosocial QoL *via* hope (αβ = −0.099, 95% CI = −0.218 to −0.019), but its effect on psychosocial QoL *via* social support was not significant (αβ = −0.160, 95% CI = −0.386 to 0.004).

**TABLE 3:** Indirect effects from hope, social support, and mRS to QoL *via* perceived stress and depression.

Time 0 predictor	Mediators	Time 2 outcome	Indirect effect	95% CI
**Individual indirect effects:**				
Hope	Time 1 Perceived stress →	Physical QoL	0.169*	0.013 to 0.376
Hope	Time 1 Depression →	Psycho QoL	0.125*	0.003 to 0.345
Social support	Hope → Time 1 Perceived stress →	Physical QoL	0.074*	0.005 to 0.171
Social support	Hope → Time 1 Depression →	Psycho QoL	0.055*	0.001 to 0.148
**Total indirect effects:**				
mRS	Social support →	Physical QoL	−0.256*	−0.621 to −0.002
mRS	Social support →	Psycho QoL	−0.160	−0.386 to 0.004
mRS	Hope →	Physical QoL	−0.139*	−0.286 to −0.029
mRS	Hope →	Psycho QoL	−0.099*	−0.218 to −0.019

CI, confidence interval; mRS, modified Rankin Scale; Psycho QoL, Psychosocial quality of life. *Significant with 95% bootstrapped confidence intervals excluding zero.

### Moderating effects of functional impairment

**FIGURE 2:**
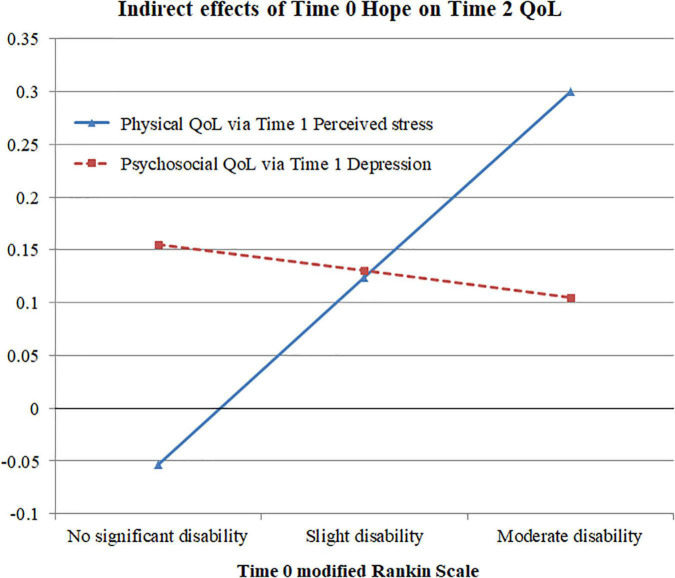
Indirect effects of Time 0 hope on Time 2 physical and psychosocial quality of life among participants with various degrees of functional impairment.

Latent interaction analysis was conducted by modeling the interaction term between functional impairment and the latent factors (social support and hope). The interaction term between functional impairment and social support did not significantly predict hope (*B* = 0.08, SE = 0.06, *p* = 0.18). The interaction term between functional impairment and hope had a significant effect on perceived stress (*B* = −0.89, SE = 0.36, *p* = 0.014) but not on depression (*B* = 0.12, SE = 0.18, *p* = 0.51) at Time 1. Figure 2 depicts the probing of indirect effects of Time 0 hope on the Time 2 QoL measures *via* Time 1 perceived stress and depression at different levels of functional impairment. The indirect effect of hope on psychosocial QoL *via* depression remained relatively stable (0.11 to 0.16) across various degrees of disability. However, the indirect effect of hope on physical QoL *via* perceived stress showed considerable variation across the different functional impairment levels. Among participants with no significant disability, the indirect effect became non-significant. Compared with participants with slight disability, those with moderate disability showed an increased indirect effect (from 0.12 to 0.30, respectively), and this increase was significant (Δ = 0.18, SE = 0.09, *p* = 0.040).

## Discussion

This study examined the temporal effects of social support and hope on emotional distress and functional outcomes in a sample of Chinese stroke survivors. The use of structural equation modeling to represent social support and hope as latent factors eliminated the measurement error bias in the regression coefficients (38). Consistent with previous findings (22, 39–42), social support had a moderate positive effect on hope. This implies that higher perceived social support from family and friends fostered greater agency and increased positive psychological functioning among the survivors. Both social support and hope were significantly negatively correlated with perceived stress and depression. However, after controlling for baseline emotional distress, only hope showed a significant negative effect on both variables at Time 1. This result suggests that hope has a stronger influence than social support on the emotional distress of stroke survivors.

The present study revealed significant negative indirect effects of social support on perceived stress and depression *via* hope. These results agree with recent findings in which hope significantly mediated the negative relationship between perceived social support and depressive symptoms in patients with prostate cancer or central nervous system tumors (43, 44). The present sample displayed improved mental well-being in the recovery process in terms of fewer depressive symptoms at 2-month follow-up and better psychosocial QoL at 8-month follow-up. In line with previous studies (45, 46), depressive symptoms had a significant negative effect on psychosocial QoL at the 6-month follow-up. In addition, perceived stress had a significant negative effect on physical QoL at the 6-month follow-up. Subjective perceived stress has been linked to physiological functioning because it can alter the neuroendocrine cortisol response (47, 48).

Stroke survivors with greater functional impairment displayed significantly lower levels of social support and hope, which highlights the greater need for psychological interventions among this subgroup of stroke survivors. Functional impairment had a significant negative direct effect on physical QoL but not on psychosocial QoL. This could be attributable to the 8-month time lag between the Time 0 and Time 2 assessments and the greater physical impact of stroke sequelae in terms of motor and sensory deficits (49). We found a chain of significant indirect effects of functional impairment on emotional distress and quality of life that was mediated by social support and hope. Recent studies (50–52) have shown similar serial mediating roles for social support and hope in the prediction of life meaning, life satisfaction, and depressive symptoms in various patient groups.

In the present study, age, number of cohabitants, type of stroke, and duration since stroke onset were not significantly associated with the study variables. However, functional impairment and hope had a significant interaction effect on perceived stress. The negative effect of hope on perceived stress became stronger (i.e., more negative) as the degree of functional impairment increased. In particular, hope played a salient role in protecting against perceived stress among survivors with moderate functional disability. This finding highlights the importance of adding elements that can foster hope to rehabilitation programs to support patients’ psychosocial needs. This could, in turn, alleviate their emotional distress and improve their physical and psychosocial QoL. It would also be beneficial to establish social support networks through mentorship schemes (53, 54) and to encourage stroke survivors to proactively use this support as a resource and source of motivation during the recovery process.

### Study limitations

This study has some limitations. First, the non-randomized and convenience sampling of study participants could have introduced self-selection bias and there could be response biases associated with the selective attrition of survivors with better functional recovery. Caution is warranted in generalizing these results to other samples of stroke survivors before replications in future studies. Second, the present study only investigated hope and emotional distress as the mediators between social support and physical QoL. Further studies should explore the role of other potential mediators such as community participation (6) and resilience (55) in leading to better functional recovery. Third, most of the data were collected *via* self-report measures and recall bias and method variance may have reduced the validity of the results (56). Future research could incorporate alternative measures such as clinical assessments and independent ratings and explore the physiological effects of social support and hope on prognostic markers of stroke recovery, such as cortisol (57, 58) and C-reactive protein (59, 60).

Fourth, social support and hope were measured at Time 0. There could be a reciprocal effect from hope to social support whereby survivors who feel more hopeful perceive greater levels of social support from their family and friends. Panel studies are needed to elucidate the causal direction between social support and hope. Fifth, the present sample comprised stroke survivors aged between 18 and 64 years. There could exist age differences in the long-term prognosis between the younger stroke survivors (between 18 and 44 years) and the middle-aged group (between 45 and 64 years) in terms of brain chemistry and psychosocial functioning (61, 62). A previous study (63) has found greater symptoms of depression but lower degrees of executive dysfunction among young stroke survivors than older patients. Further studies of larger sample sizes are recommended to test whether stroke survivors in different age groups would be affected by functional disabilities to different extents.

## Conclusion

This longitudinal study improved our understanding of the potential mediating roles of social support and hope in the functional recovery of stroke survivors. Hope appeared to play a key predictive role in mediating the effects of functional impairment and social support on emotional distress and physical and psychosocial QoL over 8 months. Future randomized clinical trials could investigate the effectiveness of social support interventions (64) and hope-based interventions (28) in improving physical and psychosocial well-being.

## Data availability statement

The raw data supporting the conclusions of this article will be made available by the authors, without undue reservation.

## Ethics statement

The studies involving human participants were reviewed and approved by the Human Research Ethics Committee, The University of Hong Kong (Reference number: EA1702058), and the Institutional Review Board of the University of Hong Kong/Hospital Authority Hong Kong West Cluster (UW18–467) and East Cluster (HKECREC-2019-111). The patients/participants provided their written informed consent to participate in this study.

## Author contributions

TF: conceptualization, formal analysis, investigation, methodology, software, validation, and writing – original draft. TL: data curation, investigation, project administration, and writing – review and editing. RH: resources, methodology, funding acquisition, software, supervision, and writing – review and editing. All authors contributed to the article and approved the submitted version.
